# Reaching the unreached: effectiveness and satisfaction with community-directed distribution of sulfadoxine-pyrimethamine for preventing malaria in pregnancy in rural South-East, Nigeria

**DOI:** 10.1186/s12936-020-03468-2

**Published:** 2020-11-07

**Authors:** Ijeoma Nkem Okedo-Alex, Ifeyinwa Chizoba Akamike, Chihurumnanya Nwachi Alo, Adaoha Pearl Agu, Chinyere Benedicta Nzeh, Chinwendu Daniel Ndukwe, Odii Ogonna Okoro, Dejene Derseh Abateneh, Chigozie Jesse Uneke

**Affiliations:** 1 African Institute for Health Policy and Health Systems, Ebonyi State University, Abakaliki, Nigeria; 2 Department of Community Medicine, Alex Ekwueme Federal University Teaching Hospital Abakaliki , Abakaliki, Ebonyi State, Nigeria; 3 Menelik II College of Medicine and Health Sciences, Kotebe Metropolitan University, P.O.Box 3268, Addis Ababa, Ethiopia

**Keywords:** Malaria, Pregnancy, Sulfadoxine-pyrimethamine, Effectiveness, Community-directed distribution, Satisfaction, Nigeria

## Abstract

**Background:**

Innovative community strategies to increase intermittent preventive treatment with sulfadoxine-pyrimethamine (IPTp-SP) coverage is advocated particularly in rural areas, where health infrastructure is weakest and malaria transmission highest. This study involved proof-of-concept implementation research to determine satisfaction with and effectiveness of community-directed distribution of IPTp-SP on uptake among pregnant women in Ebonyi State, Nigeria.

**Methods:**

This before-and-after study was carried out in 2019 in a rural community in Ebonyi State Nigeria. The intervention involved advocacy visits, community-wide sensitizations on malaria prevention, house-to-house directly observed IPTp-SP administration, and follow-up visits by trained community-selected community-directed distributors (CDDs). Monthly IPTp-SP coverage was assessed over 5 months and data analysed using SPSS version 20.

**Results:**

During the study, 229 women received the first dose of IPTp while 60 pregnant women received 5 or more doses of IPTp. The uptake of ≥ 3 IPTp doses increased from 31.4% before the community-directed distribution of IPTp to 71.6% (P < 0.001) by the fourth month post-initiation of the community-directed distribution of IPTp. Sleeping under insecticide-treated net (ITN) the night before the survey increased from 62.4 to 84.3% (P < 0.001) while reporting of fever during pregnancy decreased from 64.9 to 17.0% (P < 0.001). Although antenatal clinic utilization increased in the primary health centre serving the community, traditional birth attendants and patent medicine vendors in the community remained more patronized. Post-intervention, most mothers rated CDD services well (93.6%), were satisfied (97.6%), and preferred community IPTp administration to facility administration (92.3%).

**Conclusion:**

Community-directed distribution of IPTp-SP improved uptake of IPTp-SP and ITN use. Mothers were satisfied with the services. The authors recommend sustained large-scale implementation of community-directed distribution of IPTp with active community engagement.

## Background

Malaria infection during pregnancy is a significant public health problem with substantial risks for the pregnant woman, her fetus and the newborn child [1]. In combination with the Democratic Republic of Congo, Nigeria contributes up to 40% of the global burden of malaria [2]. Malaria contributes to an estimated 11% of maternal mortality, 25% of infant mortality and 30% of under-five mortality in Nigeria. In Ebonyi State, the leading cause of ill health and death is malaria, accounting for over 35% of mortality and more than 60% of morbidity [3].

As part of its three-fold package of interventions for the prevention and treatment of malaria in pregnancy, the World Health Organization (WHO) recommends intermittent preventive treatment in pregnancy with sulfadoxine-pyrimethamine (IPTp-SP) as part of antenatal services in areas of moderate to high transmission of *Plasmodium falciparum*. At least 3 doses of IPTp-SP should be given at antenatal care visits starting as early as possible in the second trimester up to the time of delivery, with at least a month interval between doses [1]. This has been adopted and incorporated into the Nigerian national policy on malaria prevention and control during pregnancy [4]. IPTp reduces maternal malaria episodes, maternal and fetal anaemia, placental parasitaemia, low birth weight (LBW), and neonatal mortality [1].

Despite global and national policies on IPTp-SP, coverage of this preventive treatment remains low as only 36 and 14% of rural Nigerian pregnant women received 2 doses and 3 or more doses of IPTp-SP, respectively, in 2018 [5]. The administration of IPTp has traditionally remained facility-based despite the sub-optimal utilization of antenatal care services as only 46% of pregnant women in rural Nigerian communities attend at least four antenatal care (ANC) visits [5].

Innovative community strategies that leverage existing community structures to increase IPTp coverage have been advocated, particularly in rural and remote areas where health infrastructures tend to be the weakest and malaria transmission the highest. This was highlighted as a core research priority area in the Nigerian National Malaria Operations research agenda 2017–2020 [6]. This study was a proof-of-concept implementation research to determine the effectiveness, satisfaction and challenges of community-directed implementation of IPTp in a rural Nigerian community.

## Methods

### Study area

This study was conducted in the Ebiriogu community, which is located in the Okuzzu-Ukawu political ward in Ukawu Development Centre in Onicha local government area (LGA) of Ebonyi State, Southeast Nigeria. Ukawu Development Centre has 3 political wards: Okuzzu-Ukawu, Isinkwo and Abomege. Each political ward has a variable number of primary health centres (PHCs). Okuzzu-Ukawu political ward has 6 PHCs (one of which is located in Ebiriogu) and a dispensary. Ebiriogu community has 3 settlements and one PHC, which is the major source of orthodox health care services in the community. People of the community also access health services in the PHCs located in the other political wards, as well as from traditional healers. The people of Ukawu are mostly Ibos, the dominant tribe of South-East geopolitical zone of Nigeria and their major occupations include farming and trading. Ebonyi State is located in South-East Nigeria with Abakaliki as the state capital. There are 3 senatorial zones and 13 LGAs in the state. According to the 2006 population and housing census, the population of Ebonyi State is approximately 2,176,947 with a landmass of 5,935 sq km. Infants (children < 1 year old) make up 4% of the population, children under 5 years 20%, and women of childbearing age 22% of the population [3]. Malaria transmission in Nigeria is perennial, with seasonal peaks in March to September in the south and August to November in north. Temperature and rainfall variations could affect the distribution of mosquitoes and in turn influence the seasonality of malarial episodes and symptoms [2, 7]. This study was conducted during the rainy season (June-October), which represents the seasonal peak period for malaria transmission in southern Nigeria.

In Ebonyi State, some PHCs are selected and supported by development partners while others are not. This support is usually in line with development partner’s organizational objectives and could range from capacity building on different aspects of health, monitoring and evaluation, supportive supervision, and community-level activities, among others. Ebiriogu community was selected because the PHC is not supported by any development partner. This is because, for supported facilities, development partners may have maternal health-related activities (inclusive of prevention and care for malaria in pregnancy) in the facility and community, which may confound findings from this study. Additionally, it is hoped that using a community with non-supported facilities will discourage dependence on external partners and promote sustainability, given recent donor fatigue in Nigeria and other developing countries.

### Study population

Eligible women who were in the second trimester of pregnancy, had experienced quickening and had not received a dose of SP in the previous one month. Pregnant women with a history of allergy to sulfur drugs, unexplained recurrent jaundice, or who were already on cotrimoxazole prophylaxis were excluded from receiving IPTp-SP.

### Study design

The study was an intervention study without control or randomization conducted in three phases: baseline, implementation and post-implementation evaluation.

### Data collection methods

At baseline, uptake of IPTp was assessed using interviewer-administered questionnaires among 242 pregnant women and women who had given birth within 6 months before the survey. The questionnaires were administered by trained graduate research assistants. The respondents were recruited from the PHC in Ebiriogu community as well as 4 other PHCs in Ukawu Development Centre offering immunization and antenatal care services. These other PHCs were selected based on high levels of patronage by mothers. Baseline data collection was conducted over a 3-week period. At the PHC facility, registers were used to collect data on IPTp uptake.

## Intervention

### Community-directed distributor training

The intervention included advocacy visits and stakeholder engagements with stakeholders in the community such as Ward Development Committee (WDC) chairmen and members, community and opinion leaders (traditional heads, women group leaders, market leaders, religious leaders, PHC officer-in-charge (OIC), town union leaders, youth leaders, opinion leaders). The community leaders were encouraged to select two trusted and acceptable female volunteer Community Directed Distributors (CDD) of IPTp-SP per settlement in the community. The CDDs were selected based on being trustworthy and well-motivated individuals with at least junior secondary school education who lived and worked in the community. They should also live and/or work in easily accessible sites where pregnant women can access them for IPTp-SP and other concerns. Priority in the selection of CDDs was given to women with prior childbearing experience in order to ensure the selection of CDDs acceptable to the women.

The CDDs were trained on basic information about pregnancy, malaria, malaria in pregnancy, estimation of gestational age, eligibility for IPTp-SP administration and side effects, proper use of insecticide-treated nets (ITN), counselling of pregnant women, referral to the PHC, interviewing technique, and documentation using summary forms. The training module was adapted from the National Guidelines and Strategies for Malaria Prevention and Control during Pregnancy [4]. The training was held for 3 days in the community after which the CDDs were given tool-kit bags containing client visitation forms, registers, ANC referral forms, and IPTp drugs. The training was conducted by the principal investigator and the OIC of the PHC. Before the training, the OIC received refresher training on current WHO recommendations for ANC attendance and frequency of IPTp administration. Weekly and two-weekly supportive supervision of the CDDs was conducted by the OIC and research team, respectively.

The CDDs identified the pregnant women in the community, provided general counselling on pregnancy care, including use of ITN and health-seeking for malaria symptoms to pregnant women and their family members available during the visits, administered IPTp-SP to eligible women, and referred them for ANC for prenatal care and receipt of ITNs over a 5-month period. They also followed up the pregnant women using home visits in order to encourage ANC attendance and ITN use. The CDDs carried out community distribution dressed in branded T-shirts, caps, and bags with educative pictures and write-ups on prevention of malaria in pregnancy. The CDDs received monthly financial token stipends for their transportation and meals. The drug supply to CDDs was linked to the PHC in the community and was only obtained from the facility. For the period of the intervention, the CDDs issued referral forms to pregnant women who received IPTp but were either not enrolled or poorly adherent to ANC. On accessing services in the health facility, the pregnant women were instructed to present the referral forms in addition to verbally communicating that they had been referred by CDDs in the community. With or without the referral forms, the facility health workers also directly enquired from the pregnant women who utilized the health facility whether they had been encouraged to do so through the intervention and if the response was in the affirmative, they indicated this by a tick beside the details of the women in the ANC register. Verbal confirmation was strongly emphasized because the feedback from the CDDs was that some of the women forgot to go along with their referral forms to the health facility.

Review meetings were held on a two-weekly basis with the CDDs. During the review meetings, drug stock and data collection documents were reviewed, field experiences and challenges shared and addressed. Every woman who received IPT from the CDDs was given a card on which doses and the dates the IPT were given was marked and this was presented whenever she visited a health facility for ANC or was due for another dose, in order to avoid inappropriate multiple dosing. The CDDs also visited with their records of IPT administration and verified that pregnant women had not received IPTp in the 4 weeks preceding the current administration.

### Community sensitization

A community awareness campaign was used to sensitize community members on general malaria prevention and specifically the prevention of malaria in pregnancy. The sensitization was held in the community hall and involved brief health talks, question and answer sessions, and distribution of information and education fliers on prevention of malaria. The leaflets contained pictures and short write-ups in English and Ibo languages conveying information on the prevention of malaria in pregnancy and other preventive practices. The health talks were given in the local dialect by the principal investigator and OIC of the PHC. Additionally, platforms and meetings of social groups in the community and church-based women’s groups and community political groups (tradtional cabinet, consultation meetings) were utilized to educate community members. The community town criers were also engaged to disseminate specific messages on the prevention of malaria in pregnancy.

### Post-implementation of the intervention

Over the 5 months of the intervention, a monthly implementation evaluation was conducted starting from the first month of the intervention to assess the proportion of women who received various doses of IPTp-SP using records from CDDs and ANC registers. Change in ANC attendance following the intervention was also computed from the PHC. Satisfaction with the community-directed distribution of IPTp and the CDD services was also assessed.

### Sample size determination

The estimated annual population of pregnant women in the Ebiriogu community calculated as 5% of the total population in the community was 303, as obtained from the Ebonyi State Primary Health Care Development Agency. Since the study was conducted over 5 months, about half of this number (152 pregnant women) was used as the minimum target population for the IPTp-SP distribution.

## Data management and analysis

### Measurement of variables and statistical analysis

The independent variables include the socio-demographic and clinical characteristics of the participants (age, marital status, gestational age of pregnancy, presence of quickening, parity, history of SP administration within the previous four weeks, antenatal attendance). The dependent variables were the proportion of women who received different doses of IPTp-SP, ITN use, and satisfaction with CDD services. The client visitation forms were used to collect information on the socio-demographic and clinical characteristics, ITN use and the IPT dose provided to each pregnant woman. These were then summarized every month using the monthly summary forms. Post-intervention, a short questionnaire was used to assess satisfaction with the CDD services among women who had received the IPTp.

Frequencies and proportions were calculated for categorical variables while means and standard deviations were calculated for quantitative variables. Pre-intervention and post-initiation of the intervention, proportions were compared using Chi-square. The level of significance was set at P < 0.05 and the confidence interval at 95%. The fourth month following the initiation of the intervention was used to compare with baseline ANC use, ITN ownership and use, and fever during pregnancy. The fourth month was chosen to allow the pregnant women to receive a minimum of 3 doses (in months 1–3 in line with the WHO recommendation) and also to allow time for the uptake of the intervention. The fourth month was also the month in which the highest number of women received IPTp-SP.

The IBM Statistical Package for Social Sciences (SPSS) version 20 was used for data entry and analysis. Frequency tables and figures were used to present the study findings.

## Results

The mean age of the study participants was 25.8 ± 5.0 yeaers and the majority (77.0–93.0%) had attended at least one ANC visit. In the first month of the initiation of the intervention, 55.9% of the women had received IPTp and this increased during the intervention months. The CDDs were the major source of IPTp (33.5–78.1%) across the months of the intervention. Net ownership (82.3–92.3%) and sleeping under the net the night before the survey (81.0–91.6%) remained high during the survey (Table 1).

**Table 1:** Monthly socio-demographic, antenatal and malaria-related characteristics of the respondents during the study intervention period. Data source: Client visitation and monthly summary forms

Variable	Month 1 n = 143 (%)	Month 2 n = 158 (%)	Month 3 n = 153 (%)	Month 4 n = 230 (%)	Month 5 n = 155 (%)
Mean age ± SD	25.8 ± 5.0				
At least one ANC visit	111 (77.6)	147 (93.0)	127 (83.0)	177 (77.0)	136 (87.7)
Mean number of ANC attended	2.9 ± 1.4 (n = 111)	3.0 ± 1.8 (n = 147)	3.1 ± 1.7 (n = 127)	3.2 ± 1.5 (n = 177)	3.0 ± 2.1 (n = 155)
Ever received IPTp	80 (55.9)	105 (66.5)	130 (85.0)	164 (71.3)	133 (85.8)
Source of IPTp					
PHC	55 (38.5)	40 (25.3)	18 (11.8)	31 (13.5)	12 (7.7)
CDD	0 (0.0)	53 (33.5)	108 (70.6)	127 (55.2)	121 (78.1)
Patent medicine vendor	24 (16.8)	12 (7.6)	4 (2.6)	6 (2.6)	0 (0.0)
Others	1 (0.7)	1 (0.6)	0 (0.0)	0 (0.0)	0 (0.0)
ITN Ownership	121 (84.6)	130 (82.3)	130 (85.0)	195 (84.8)	143 (92.3)
Slept under ITN the night before survey	118 (82.5)	128 (81.0)	129 (84.3)	194 (84.3)	142 (91.6)
Had a fever in this pregnancy	62 (43.4)	44 (27.8)	27 (17.6)	39 (17.0)	33 (21.3)
Treated for malaria in this pregnancy	56 (39.2)	73 (46.2)	35 (22.9)	60 (26.1)	31 (20.0)

During the study, the number of pregnant of women who received different doses of IPTp was as follows: one dose (n = 229), 2 doses (n = 232), 3 doses (n = 217), 4 doses (n = 121), 5 doses (n = 34), and 6 doses (n = 6) (Table 2).

**Table 2:** IPTp (IPT1-1PT6) uptake by month during the study intervention period. Data source: Monthly summary forms

IPT doses	Month 1	Month 2	Month 3	Month 4	Month 5	Total
IPT 1	65	53	23	66	22	229
IPT 2	34	48	40	59	51	232
IPT 3	28	43	61	43	42	217
IPT 4	15	14	24	49	19	121
IPT 5	1	0	5	11	17	34
IPT 6	0	0	0	2	4	6
Total	143	158	153	230	155	

At baseline, 23.1% of respondents received IPT 1 and this increased to 75.6% post-intervention (P < 0.001). At baseline, the uptake of 4 or more doses of IPTp was 12.8% and this increased to 53.1% post-intervention (P < 0.001) (Table 3).

**Table 3:** Doses of IPTp-SP taken during pregnancy at baseline and in the fourth month of the intervention. Data source: Baseline survey and client visitation and summary forms in the fourth month post-initiation of community-directed distribution of IPTp

IPT Dose	Baseline	Month 4 of the intervention	χ^2^ (P value)
	n = 242	n = 303	
	n (%)	n (%)	
IPT 1	56 (23.1)	229 (75.6)	148.3 (< 0.001)^a^
IPT 2	44 (18.2)	232 (76.6)	183.5 (< 0.001)^a^
IPT 3	76 (31.4)	217 (71.6)	87.4 (< 0.001)^a^
IPT 4 or more	31 (12.8)	161 (53.1)	95.9 (< 0.001)^a^

^a^Statistically significant

The proportion of respondents who slept under ITN increased from 62.4 to 84.3% (P < 0.001) while the reporting of fever during pregnancy decreased from 64.9 to 17.0% (P < 0.001) at post-intervention. Having at least one ANC visit decreased by 17.2% when compared at baseline and fourth month of the intervention (P < 0.001) (Table 4).

**Table 4:** Access to antenatal care and malaria-related characteristics at baseline and in the fourth month of the intervention. Data source: Baseline survey and client visitation and summary forms in the fourth month post-initiation of community-directed distribution of IPTp

Variable	Baseline	4th month of the intervention	χ^2^ (P-value)
	n= 242	n = 230	
	n (%)	n (%)	
At least one ANC visit	228 (94.2)	177 (77.0)	28.8 (< 0.001)^a^
ITN ownership	192 (79.3)	195 (84.8)	2.36 (0.124)
Slept under ITN a night before the interview	151 (62.4)	194 (84.3)	28.9 (< 0.001)^a^
Fever while pregnant	157 (64.9)	39 (17.0)	111.3 (< 0.001)^a^

^a^Statistically significant

Over half of the respondents considered CDD services to be excellent (57.0%) and were very satisfied with the community-directed distribution of the IPTp project. The majority of respondents (92.3%) preferred community-based administration of IPTp compared to the traditional facility-based strategy (Table 5).

**Table 5:** Satisfaction with the community-directed distribution of IPTp-SP and IPTp administration preferences among women who received IPTp through CDDs in the community (n = 377). Data source: Post-implementation survey at end of the fifth month among women who received IPTp through CDDs in the community

Variable	Frequency	Percent (%)
Rating of CDD services		
Excellent	215	57.0
Good	138	36.6
Average	24	6.4
Poor	0	0.0
Very poor	0	0.0
Satisfaction with CDI-IPTp project		
Very satisfied	211	56.0
Satisfied	159	42.2
Neutral	0	0.0
Unsatisfied	4	1.1
Very dissatisfied	3	0.8
IPTp administration strategy preference		
Facility-based	29	7.7
Community-based	348	92.3

**Fig. 1:**
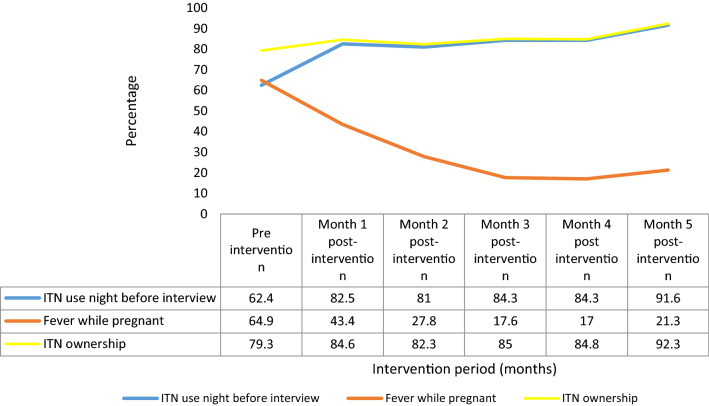
The trend of ITN use a night before the survey and occurrence of fever during pregnancy

The reporting of fever decreased while the use of ITN use the night before the survey increased during the study period. Ownership of ITN remained high during the study period with a sharp increase in the 5th month of the intervention (Fig. 1).

## Discussion

Following a community-based intervention that promoted malaria prevention in pregnancy, the findings from this study showed significant increases in the uptake of IPTp in line with the current WHO recommendation of at least 3 doses in pregnancy. This increase remained significant across the first to 4 or more doses of IPTp among women in this study. Similarly, other studies have shown that community distribution of IPTp enhanced uptake of IPTp among pregnant mothers in developing countries [8–10].

The use of an ITN the night before the survey also improved among the respondents. The fact that this intervention strategy served to reinforce the practice of sleeping under ITN in the immediate recall period is important to the design of behaviour change communication programmes targeted at promoting treated net use in pregnancy. The use and not just ownership of ITN is a core behavioural change pertinent to the control of malaria in pregnancy [11]. Furthermore, ITN use and IPTp have been found to complement each other and the use of both commodities has been found to make malaria parasitaemia less likely [12, 13]. The authors believe that this effect was observed because of the frequent personalized health education, follow-up visits and reminders by CDDs who were well-known to the mothers and could relate to their maternal health concerns being mothers themselves.

Poor antenatal uptake has been identified as a bottleneck to the effective administration of IPTp to pregnant women in developing countries where antenatal utilization tends to be sub-optimal [14, 15]. There are also missed opportunities, supply and provider level bottlenecks to the scale-up and use of interventions to control malaria in pregnancy delivered through ANC [16]. Given this, there is an increasing focus on devising implementation research strategies that address these bottlenecks and problems by promoting universal access to life-saving malaria prevention commodities, such as IPTp [4]. This is especially so for malaria-endemic countries such as Nigeria where malaria accounts for high levels of mortality and morbidity among pregnant women and under-five children [2]. Additionally, studies have shown that although IPTp drugs are given free of charge in public health facilities, women perceive IPTp services to be costly (not free) alongside other costs associated with utilizing formal health care services [17, 18]. This is typical of the rural women in this study and makes a case for the use of community-directed and based strategies in improving maternal health and specifically for preventing malaria in pregnancy because poor women are less likely to attend ANC and in turn receive IPTp from the health facility [19]. However, such community approaches as used in this study have been shown to be cost-effective and feasible [8–10].

This study revealed that there was a decrease in fever reported during pregnancy among the women exposed to the intervention. This could reflect the effect of the protection afforded using IPTp or could infer that with better knowledge of malaria symptomatology in pregnancy, the study participants were able to distinguish malaria symptoms from those of normal pregnancy. Also, since this study was conducted during the seasonal peak of malaria, this decline may have been due to increased cases of fever among pregnant women with a gradual decline over time. The authors admit that the before-after design of this study did not permit the establishment of temporality in this regard. Paradoxically, the proportion of women who reported treating malaria in pregnancy varied by month. This could be due to improved health-seeking for symptoms of malaria given the education on malaria symptomatology by the CDDs and the improved knowledge reported among the pregnant mothers [18]. The unique composition of each cohort of women identified for IPTp by the CDDs accounted for by newly pregnant women, pregnant women missed in the preceding month and those who had delivered (and left the cohort) could explain the variations in the reported treatment for malaria in pregnancy.

A core concern of the community-based distribution of IPTp-SP has been that it could decrease ANC attendance [10]. This study found that ANC attendance varied across the months of the intervention with a decrease when compared with the baseline findings. A first guess would be the possible diversion of the pregnant women from receipt of antenatal services by the CDDs. This is unlikely because the CDDs provided only IPTp and basic education on malaria prevention and referred women to ANC for the full package of care. They ensured the women understood that they were not a substitute for ANC attendance. Nonetheless, tacit evidence from the community and feedback from the CDDs showed that pregnant women preferred to patronize traditional birth attendants and patent medicine vendors (PMV or ‘chemists’) for prenatal care. One of the assertions from a PHC worker was that an unintended effect of previous training of PMVs (by development partners) on malaria diagnosis using rapid diagnostic tests was that the PMVs proceeded to divert or attract patronage for ANC from pregnant women. However, this is only anecdotal evidence and requires more investigation using scientific research methods. More work still needs to be done to strengthen antenatal care utilization given the questionable content of the antenatal care package likely to be obtained from such non-skilled antenatal care providers. There was an underlying culture-related preference for traditional medical care, including prenatal care, among women in the community and this could affect orthodox antenatal care-seeking behaviour. From previous literature, there have been mixed findings regarding the effect of such community-directed interventions on antenatal care use. While some studies had found increased antenatal care utilization [10], some have documented no effect [8] or decreased ANC usage [20]. The results of this study should be interpreted with caution because of the difference in the respondents at baseline (pregnant women and women who had given birth within 6 months prior to the survey) and post-initiation of the intervention (pregnant women only). The poor quality of the records from the health facilities in the community did not permit objective quantification although the facility managers subjectively affirmed increased antenatal attendance.

The majority of women expressed satisfaction with the community-directed distribution of IPTp rendered by the CDDs. The women preferred community administration over facility administration. The CDDs reported that women eagerly looked forward to the next dose reminding them of chance meetings, the men asked that they are given such similar medical attention while generally community members recognized them (by the branded materials) during their visits. Patient satisfaction with care is expected to translate into better utilization and compliance with health services in the future and is useful in the planning and evaluation of health programmes [21, 22].

The extent to which the findings of this study can be generalized to urban settings and different contexts is limited because it was conducted in one community in Nigeria. The absence of a control arm may affect the conclusions that the intervention led to an improvement.

## Conclusion

In this study, the community-directed distribution of IPTp-SP improved uptake of IPTp-SP and ITN use. There was decreased reporting of fever during pregnancy. The study further established that antenatal care utilization increased following the intervention. Most of the study participants rated the CDD services highly, were satisfied with the project, and preferred the community-directed distribution over facility-based administration of IPTp. The authors recommend sustained large-scale implementation of community-directed distribution of IPTp with the active engagement of the community.

## Data Availability

The datasets used and/or analysed during the current study are available from the corresponding author on reasonable request.

## Abbreviations

ANC: Antenatal care
CDDs: Community directed distributors
IPTp: Intermittent preventive treatment in pregnancy
IPTp-SP: Intermittent preventive treatment with sulfadoxine-pyrimethamine
ITN: Insecticide-treated net
LBW: Low birth weight
LGAs: Local government areas
PHC: Primary health centre
WDC: Ward development committee
WHO: World Health Organization
